# Multistability and metastability: understanding dynamic coordination in the brain

**DOI:** 10.1098/rstb.2011.0351

**Published:** 2012-04-05

**Authors:** J. A. Scott Kelso

**Affiliations:** 1 Human Brain and Behavior Laboratory, Center for Complex Systems and Brain Sciences, Florida Atlantic University, Boca Raton, FL 33435, USA; 2 Intelligent Systems Research Centre, University of Ulster, Derry, UK

**Keywords:** coordination dynamics, metastability, multistability, instability, transitions, complementarity

## Abstract

Multistable coordination dynamics exists at many levels, from multifunctional neural circuits in vertebrates and invertebrates to large-scale neural circuitry in humans. Moreover, multistability spans (at least) the domains of action and perception, and has been found to place constraints upon, even dictating the nature of, intentional change and the skill-learning process. This paper reviews some of the key evidence for multistability in the aforementioned areas, and illustrates how it has been measured, modelled and theoretically understood. It then suggests how multistability—when combined with essential aspects of coordination dynamics such as instability, transitions and (especially) metastability—provides a platform for understanding coupling and the creative dynamics of complex goal-directed systems, including the brain and the brain–behaviour relation.

## 1. Introduction

In addressing the subject of multistability, we are advised to follow Socrates: first ask what? Then ask why? So, what is multistability and how do we understand it? In previous work, my colleagues and I have considered multistability and its cognate aspects (fluctuations, instability, transitions, metastability, hysteresis, adaptation, etc.) in vision, speech, language, motor and neural dynamics ([1]; see also earlier studies [2–4] for related approaches). In the last 15 years, many empirical generalizations and modelling developments have taken place on the subject, at least in part owing to the advent of structural and functional neuroimaging and increasingly sophisticated analysis and computational modelling tools. Here, however, after a few general words on what multistability is, I wish to focus on the question of *why* multistability occurs in the first place. For that one has to go beyond traditional disciplinary boundaries and fully embrace the science of complex systems tailored to the goal-directed and functional aspects of living things—coordination dynamics—where multistability is not some freak phenomenon [5], but rather is close to the very core of the way things are. The hope is that asking the why question may reshape our perspective by providing not only a language for understanding multistability but also a rationale for its occurrence, indeed its ubiquity.

## 2. What is multistability?

Multistability is a universal, essentially nonlinear aspect of matter and its organization—from molecular arrangements and chemical reactions to multistability in the meaning of words and actions and beyond [6]. When we talk of multistability, we are usually talking about stable states or attractors; the stability of a state depends on how quickly the system returns to a state following a perturbation.^1^ In the brain, attractors correspond to stable patterns of reverberating activity in neural populations that support and sustain themselves. The image is one of a changing dynamic landscape where population activity shifts from one attractive state to another. Fluctuations in the nervous system occur on many levels, from channel noise in synaptic transmission to neural populations and neural networks ([7] for review), destabilizing self-sustaining patterns and causing switching from one attractor state to another. A specific example comes from recent biophysical modelling [8] in which multistability and scale-invariant fluctuations arise in resting state cortical activity as a result of noisy input into thalamic neurons modulated by cortical feedback. In short, if a system has multiple coexisting attractors and noise is sufficiently strong to cause switching among stable states, it may be said to be multistable.

Understanding multistability may proceed in (at least) two ways. In the first, one may seek specific *mechanisms* for a given instance of multistable phenomena. The search for specific mechanism proceeds by identifying the physico-chemico-bio-psycho- basis of a chosen exemplar of interest. A second way is to seek dynamic *laws* and principles of multistability. In this approach, the same dynamic laws of multistability are expected to exist across multiple scales of observation regardless of their specific mechanistic realization. Such a ‘law-based’ perspective leads us to notice parallels between different expressions of multistability across a wide range of systems, functions and timescales, seeing the connections between them and trying to uncover the functional roles that multistability may play [9,10]. As we shall see, multistability appears in unexpected guises, playing a central role where we might not even expect it to such as in skill learning and intentional change.

Universal dynamics springs from inferred principles, specific mechanisms from close examination of particular cases. Dynamical laws and specific mechanisms can thus be seen as complementary [11]: both are needed for a comprehensive understanding of multistability and cognate phenomena. This apparent tension between dynamics and mechanism may be seen as an advantage: universal dynamics allows us to see the connections across manifold expressions of multistability in a level- and mechanism-independent way. At the same time, we need particular realizations if we are ever to extract general laws and principles [12], thereby focusing our attention on mechanisms. It should be noted that many physicists take the word mechanism to mean that dynamical laws at one level are connected to, if not to be eventually replaced by, dynamical laws at lower levels (as in the case of quantum mechanics). Whether one accepts it or not, such a perspective does not deny the possibility of eventually identifying universal (dynamical) mechanisms for multistability and related phenomena across scales and levels of observation. The present contribution may be seen as a move in that direction.

## 3. Why multistability?

Why are systems, particularly complex biological systems (including, but not necessarily restricted to the brain) composed of very many interacting parts and processes and capable of producing a large repertoire of coordinated patterns of behaviour, multistable in the first place? Any answer to the question ‘why multistability?’ is likely to be complicated and have many layers, yet—viewed from the perspective of coordination dynamics—appears to involve a sequence of steps. In what follows, degeneracy and multifunctionality are proposed as fundamental aspects of living things (step 1) inextricably tied to the emergence of synergies (step 2) which are shown to be formed by self-organizing processes (step 3). The basic mechanism of self-organization is the non-equilibrium phase transition, the resulting dynamics of which gives rise to multistability (step 4). In step 5, some useful modelling tools are used to illustrate multistability and switching in behaviour and the brain, including learning (step 6). Step 7 goes beyond multistability and discusses why metastability is crucial to understanding brain and cognitive function. In this light, multistability appears as just one ingredient of a series of phenomena in which progressive coupling and broken symmetry between individual heterogeneous components enable switching from constant ‘invariant’ behaviour to creative dynamics. Metastability thus plays the role of a pivot towards the emergence of stable collective states, whether monostable or multistable. Finally, we propose that the weak coupling and broken symmetry that give rise to metastability are both the basis of and based upon the principle of complementarity [9,11].

### (a) Step 1: degeneracy

First and foremost is the need to recognize that complex biological systems at all relevant scales are degenerate. Degeneracy means that at every conceivable level of description, the same outcome or function can be achieved in many ways using different components. Edelman & Gally [13] provide evidence of degeneracy from the level of the genetic code (where multiple gene products contribute to almost any behaviour or function and every gene has the potential for pleiotropy) to social communication (where there are a near infinite number of ways to communicate the same message). In perception, many different stimulus configurations can give rise to the same percept (‘invariance’). In movement, many different muscle configurations can produce the same outcome. The same network activity in central pattern-generating circuits can be produced using many different combinations of synaptic strengths and neuron properties ([14]; see also Cymbalyuk *et al*. [15]). I have called this ‘The principle of functional equivalence’ [16] because the principle appears to operate at every level of biological organization. The complement of degeneracy or functional equivalence is *multifunctionality*: the same anatomical structures can play multiple functional roles. For example, fronto-parietal regions of the brain are involved in many other executive functions beyond their ‘top-down’ influences in multistable perception [17]. The same neural circuitry even in lowly creatures such as *Caenorhabditis elegans* [18] or the stomatogastric ganglion of the lobster [19] participates in multiple functions. Degeneracy and multifunctionality imply that there is no one-to-one mapping between structure and function. On the contrary, from gene networks to neural networks to social networks, degeneracy and multifunctionality rule: multifunctional circuit mechanisms are conserved across vertebrate and invertebrate species [19]. From the perspective of coordination dynamics, multifunctionality and degeneracy/functional equivalence can be understood in terms of multistability [20,21].

### (b) Step 2: synergies

Edelman & Gally [13] provide compelling evidence and argument that degeneracy is both a prerequisite for and an inescapable product of natural selection. But Nature has another mechanism to generate degeneracy not considered by Edelman: it synergizes. Synergies are exquisitely context-sensitive *functional* groupings of elements that are temporarily assembled to act as a single coherent unit [22,23]. Depending on context, synergies may accomplish different functions using some of the same components (e.g. the jaw, tongue and teeth to speak and chew) and the same function using different components (e.g. ‘hand’ writing with a pen attached to the big toe). Operationally, the hallmark of a synergy is that during the course of ordinary function a perturbation to any part of the synergy is immediately compensated for by remotely linked parts in such a way as to preserve functional integrity. As a complex system composed of billions of cells and synapses which in turn is capable of displaying a complex repertoire of coordinated behaviours, the brain is likely to be highly synergized. To identify brain synergies, it would be necessary to perturb one member of the synergy (e.g. a piece of cortical tissue known to be engaged for a given task or function) and observe remote and near-immediate compensation by other putatively linked brain areas. A modern tool such as transcranial magnetic stimulation (TMS) [24] when combined with sophisticated imaging technologies to record remote effects may be a way to discover synergies in the human brain. There are probably other techniques such as microstimulation (L. Ungerleider 2010, personal communication).

### (c) Step 3: self-organization and dynamic instability

The basic mechanism for the formation and change of synergies—complementing natural selection—is self-organization [9,10,23], a mechanism (not known to Darwin) that Nature uses to form spatial and temporal patterns in non-equilibrium systems that are open to exchange of energy, matter and information with their environments [25–28]. The most primitive form of self-organization is the non-equilibrium phase transition, which appears near the so-called critical ‘tipping’ points or instabilities when a control parameter crosses a threshold. Close to instability, the individual elements, in order to accommodate current conditions, order themselves in new or different ways: the faster (typically microscopic) individual elements or variables in the system become ‘enslaved’ to the slower (typically macroscopic) collective variables [25]. The macroscopic patterns that emerge may be defined as stable attractive states of the collective variable dynamics. Fluctuations are always present, testing whether a given pattern is stable and allowing the system to discover new, more adaptive patterns (for a related view called *self-organized criticality* see Chialvo [29] and Plenz & Thiagarian [30] for reviews). Why is the non-equilibrium phase transition—which can take many forms in nature—so important for our present concerns? The answer is that (i) it is a fundamental mechanism for pattern formation in open systems; (ii) used as a methodology, it enables the identification of relevant (collective) variables and their dynamics in complex systems—which in neuroscience are often not known a priori; and (iii) the non-equilibrium phase transition qua dynamic instability (whether a switch from randomness to order and vice versa or from one ordered state to another) implicates multistability as an inherent aspect of nature and of tremendous selective value [31].

### (d) Step 4: multistable coordination dynamics

Non-equilibrium phase transitions have been demonstrated to be the basic self-organizing mechanism for the assembly and formation of functional synergies [20,21]. Consider some basic forms of coordination from a variety of complex systems studied in laboratory settings. Whether coordinating the movements of the limbs, whether coordinating individual body parts with tactile, visual or auditory stimuli, whether two people are coordinating together, spontaneously or intentionally, whether a human is coordinating with an avatar [32] or even riding a horse [33], just two stable patterns among the interacting components and processes predominate: in-phase and anti-phase (see earlier studies [10,34–36] for reviews). Despite (or perhaps because of) the numerous neurons, muscles, joints and metabolic processes involved in these coordinations, despite the numerous differences between the parts and processes that are doing the coordinating and the multitude of ways the parts can be coupled with each other and with the environment, all these different systems have been demonstrated to exhibit *bistability*, the simplest form of multistability.

To better understand how bistability originates at phase transitions requires one to use some concepts, methods and formal tools that we can only mention briefly here [20,21,37,38]. Key ones concern the identification of collective variables, the control parameters that move the system through its collective states and the minimum form taken by the dynamics. Many variables are changing in the experimental circumstances described above, but one that captures the essence of the coordination patterns under scrutiny here and changes abruptly at transitions is the relative phase, *ϕ*. Notice that this quantity ‘enfolds’ different domains: much evidence shows that relative phase characterizes the synergic interaction between parts that range from neurons to body parts to people and machines. As a key coordination variable, *ϕ* spans the processes that couple stimuli and responses, perception and action, organism and environment, brain and body, and even brains acting together [39,40].

A general form of the coordination dynamics referred to as the Schöner–Kelso conjecture in Turvey [41] is:3.1
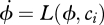
where the temporal change of the coordination variable *ϕ* (phi dot or 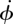) is a function of the variable itself subject to both specific and non-specific parametric influences, *c_i_*.

A particular elementary form of the coordination dynamics called the extended HKB model (after Haken, Kelso and Bunz [42]) is3.2
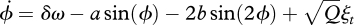
where *ϕ* is the relative phase, 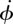 is the derivative of *ϕ* with respect to time, *a* and *b* are coupling parameters the ratio of which (*k* = *b*/*a*) is a non-specific control parameter in experiments, *δω* = *ω*_1_ − *ω*_2_ reflects intrinsic differences between the components and *ξ*_*t*_ is a stochastic noise term of strength *Q* reflecting the fact that fluctuations are present in all real systems. We say *b*/*a* is a *non-specific* control parameter because it simply moves the system through its coordinative states but does not specify or ‘encode’ them.

An equivalent form of the extended HKB equation is3.3
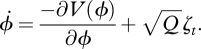


With the potential3.4
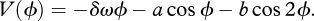
The coordination dynamics can be visualized as a particle moving in a potential function, *V*(*ϕ*). The minima of the potential are points of vanishing force, giving rise to stable solutions of the coordination dynamics. For *δω* = *ω*_1_ − *ω*_2_ = 0 and for low values of the control parameter (*b*/*a*), the system has two stable attractive coordination states at *ϕ* = 0 and *ϕ* = ±π_rad_. Thus, two coordination states coexist for exactly the same parameter values, the essentially nonlinear feature of *bistability*.

As the ratio *b*/*a* is decreased, the formerly stable fixed point at *ϕ* = ±π_rad_ becomes unstable and turns into a *repellor*. Any small transient perturbation or fluctuation will now kick the system into the *basin of attraction* of the stable fixed point at *ϕ* = 0. Notice also that once there, the system's behaviour will stay in the in-phase attractor, even if the direction of the control parameter is reversed. Such hysteresis, a basic form of memory in nonlinear dynamical systems, is exactly what is observed in experiments.^2^

**Figure 1.**
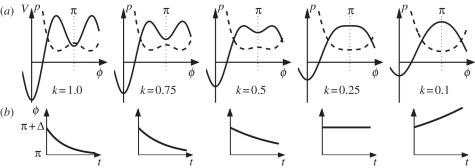
(*a*,*b*) Hallmarks of non-equilibrium phase transitions illustrated in the extended HKB model of coordination dynamics, i.e. the potential, *V*, in equation (3.4) with *δω* = 0 (solid curves in (*a*)). Enhancement of fluctuations is indicated by the widening of the probability distribution, *p*, of relative phase around *Φ* = π (dashed and dotted lines in (*a*) respectively) as the control parameter *k* = *b*/*a* decreases. Critical slowing down is revealed by an increase in the time it takes for the system to recover from a small perturbation (*b*). Note how this *relaxation time* increases as the system approaches instability. At the critical point (*k* = 0.25) and beyond, the system does not return to its former (non-equilibrium) state.

The switching from bistability to monostability as function of a control parameter is called a *bifurcation*. However, it is the presence of stochastic fluctuations that establish the existence of non-equilibrium phase transitions in biological coordination and that allow quantitative evaluation of key predictions. *Critical slowing down* is easy to understand. As the minima of the potential at *ϕ* = ±π_rad_ become shallower and shallower (figure 1*a*, solid curve, as *k* decreases) the time it takes to adjust to a small perturbation takes longer and longer. Thus, the local relaxation time (figure 1*b*) is predicted to increase as the instability is approached because the restoring force (given as the gradient in the potential, *V*(*ϕ*)) becomes smaller. Likewise, *enhancement of fluctuations* predicts that the variability of *ϕ* should increase owing to the flattening of the potential and broadening of the probability distribution, *p*, near the transition point. Both predictions have been confirmed in a wide variety of experimental systems, including magnetoencephalography and electroencephalography recordings of the human brain [44,45]. Indeed, a remarkable study by Meyer-Lindenberg *et al*. [46] demonstrated that a transition between bistable coordination patterns can be elicited in the human brain by transient TMS. As predicted by the HKB model of coordination dynamics, TMS perturbations of relevant brain regions such as premotor and supplementary motor cortices caused a behavioural transition from the less stable anti-phase state to the more stable in-phase state, but not vice versa.

That the stability of coordination states is a governing factor in how the brain works is further illustrated in a recent functional magnetic resonance imaging (fMRI) study by Jantzen *et al*. [47]. In a sensorimotor coordination paradigm, Jantzen *et al*. [47] demonstrated a clear dissociation between those neural regions that are activated when a control parameter changes and those connected to a pattern's stability. A key result was that the activation of cortical regions supporting coordination (e.g. left and right ventral premotor cortex, insula, pre-supplementary motor area and cerebellum) scaled directly with the stability of the coordination pattern. As the anti-phase pattern became increasingly less stable and more variable, so too did activation of these areas: thus it is that these parts of the brain—which form a functional circuit—have to work harder to hold coordination together. And thus it is that the difficulty of a task, often described in terms of ‘information processing load’, is captured by a dynamic measure of stability that is directly and lawfully related to the amount of energy used by the brain. Importantly, for identical control parameter values, the same brain regions do not change their activation at all for the more stable (less variable) in-phase pattern. Given the constraints, a path is chosen that tends to favour the most stable state, here the potential minimum of a collective variable, the relative phase.

The Jantzen *et al*. paper shows how multistability is realized by the same cortical circuitry which itself is exquisitely sensitive to the (in)stability of behaviour. Together with the Meyer-Lindenberg *et al*. study this work illustrates the power of coordination dynamics to predict the dynamical behaviour of the brain at the level of cortical circuitry. Dynamic stability and instability appear to be major determinants of the recruitment and dissolution of brain networks, providing both flexibility and stability in response to control parameter changes. Multistability confers a tremendous selective advantage to the brain and to nervous systems in general (cf. [18,48]): it means that the brain has multiple patterns at its disposal and can switch among them to meet environmental or internal demands. Shifting equilibria among coexisting functional states on exposure to a new set of conditions is potentially more efficient than having to create states de novo. This hypothesis can be examined further by studying how different combinations of sound, touch, vision and movement come together and spontaneously split apart in time as parameters are varied [12,49,50].

### (e) Step 5: specific parametric influences on multistable systems

Standard theories of self-organization do not incorporate goal-directedness; for that one needs to consider the interplay between multistability and *specific* parametric influences, the second cornerstone of coordination dynamics [9,10]. In the perceptual multistability literature, the latter are variously categorized in terms of top-down effects; it is well-known, for example, that perceivers can consciously switch from one interpretation of a stimulus pattern to another, and that attention and emotion can affect which alternative is selected. In coordination dynamics, intentional processes parametrize the dynamics, that is, they can stabilize or destabilize the coordination states of a multistable dynamical system. In turn, the relative stability of the coordination states dictates the nature of the intentional switching process. Both theory and data afford insight into this interplay of forces [51–54]. Experimental observations show (i) that intention can stabilize coordination states even under conditions where they would otherwise become unstable and spontaneously switch; (ii) the relative stability of the states—the intrinsic dynamics—determines how fast one state can switch to another: intentionally switching from a more stable to a less stable state takes longer than the reverse; and (iii) much greater blood oxygen level-dependent (BOLD) activity is observed in bilateral putamen when human subjects are required to switch from a more stable to a less stable state than vice versa, indicating that the basal ganglia (BG) are highly sensitive to pattern stability.

**Figure 2.**
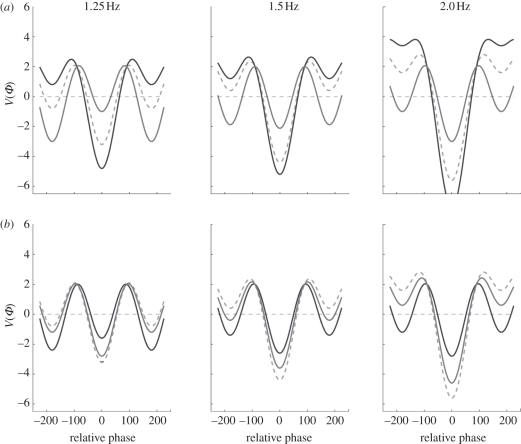
Potential plots (equations (3.4) and (3.5)) of the intrinsic (dotted grey) and intentional dynamics scaled according to basal ganglia activity prior to (black) and during pattern switching (grey; see text). (*a*) In-phase initial condition. (*b*) Anti-phase initial condition.

In the potential function of the coordination dynamics (equation (3.4)), the specific influence of intention may be represented as3.5
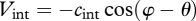
where *c*_int_ is a parameter proportional to the strength of intention and *θ* is the intended relative phase. The modified dynamics resulting from the summation of these two functions (equations (3.4) and (3.5)) is illustrated by the black and grey curves in figure 2.

The theoretical model illustrated in figure 2 operates under two assumptions. First, the level of activity in the BG represents the level of intentional ‘forcing’ or parametrization of the dynamics. This allows the BOLD estimates of putamen activity in the DeLuca *et al*. [51] experiment to be used to scale the variable *c*_int_ in equation (3.5).^3^ Second, the intended relative phase (*θ*) is assumed to follow task instructions: when participants start out in-phase (figure 2*a*), the intended pre-switch pattern (black) is in-phase and the intended post-switch pattern (grey) is anti-phase—and inversely when participants start out anti-phase (figure 2*b*). The result shows how the process of intention, stemming from the BG, is governed by the intrinsically multistable dynamics. Prior to the switch, the BG is active in both in-phase and anti-phase states. The result seen in black in figure 2*a*,*b* is to increase the stability of the required pattern. During the switch (when the intended pattern switches and *θ* goes from 0° to 180° or vice versa), activity in the BG remains high when switching from an in-phase pattern. The result is to allow for a stable anti-phase pattern as shown (in grey) across the top row. However, when switching from anti-phase to in-phase, the DeLuca *et al*. [51] experiment shows that BOLD activity in the BG dramatically decreases. The result is to release the dynamics from intentional control and thereby allow the intrinsic dynamics to accomplish the switch from the less stable (anti-phase) to the always more stable in-phase pattern. From these data we can conclude that multistable coordination dynamics places lawful constraints on what is possible to realize and what is not. It is the joint forces of intention (equation (3.5)) and the gradients defining the relative stability of the coordination states (equation (3.4)) that dictate the switching time between them—implemented in this specific example by the BG and related cortical circuitry.

### (f) Step 6: multistability in learning

Multistability plays an unforeseen but central role in learning: theoretically, it corresponds to pre-existing dispositions in an attractor landscape (intrinsic dynamics) that is shaped by learning and dictates the very nature of change. Although the hypothesis is intuitive that learning and development involve a modification of the individual's current behavioural repertoire [55], identifying and tapping into the latter has proved difficult in the extreme. To address changes owing to learning, it is necessary to devise an operational means to probe the individual learner's repertoire before, during and after the learning process. Because much is known about them, bimanual rhythmic coordination patterns in response to visual input have proved to be a useful window into skill learning [56–59]. These patterns can be changed by gradually manipulating the relevant coordination variable (here, relative phase) over its full range, thereby allowing the experimenter to probe the entire repertoire of possible patterns that subjects can produce before a learning task is introduced. This experimental procedure is called a ‘scanning probe’: in a typical experiment, two light-emitting diodes display 13 relative phases ranging from 0° to 180° in steps of 15°. Participants are required to move each index finger in coincidence with each visual stimulus. The significance of such scanning probes is that they reveal the presence of preferred behaviours—a pre-existing repertoire that constrains what can be learned and thus the very nature of the learning process.

**Figure 3.**
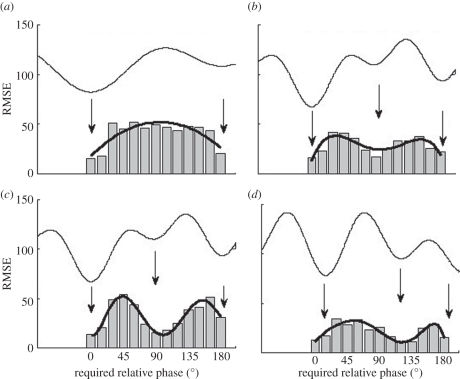
Two routes to learning. RMSE (in degrees) of the performed relative phase during scanning probes that specify a required phase before (*a*,*c*) and after (*b*,*d*) learning a new pattern. Arrows indicate minimum error corresponding to wells in potential functions (shown for illustration purposes only). (*a*,*b*) The bifurcation route in initially bistable participants: with learning two minima shift to three, a qualitative change. (*c*,*d*) The shift route in initially tristable participants: the number of minima do not change with learning (see text). Data and figure courtesy Viviane Kostrubiec (adapted from Zanone *et al*. [60]).

What does the pre-existing repertoire look like in naïve participants before learning and how is this related to multistability? In the normal adult population, scanning probes reveal two sets of results. In one sub-population, the behavioural repertoire prior to learning is composed of just two stable or preferred coordination patterns corresponding to minimum root-mean-squared error (RMSE) and illustrated by wells in a potential function shown in figure 3*a*. The attractor landscape pre-learning is thus *bistable* with attractive states located at 0° and 180°. In the other, a smaller number of individuals exhibit an additional minimum RMSE at 90° indicating that this attractive state is also part of their pre-existing repertoire (figure 3*c*). Thus, their initial dynamics is *multistable* before learning, with three potential minima.

Learning means the acquisition and persistence over time of a new pattern of behaviour. Knowledge of the pre-existing repertoire is thus instrumental in defining what a new pattern is, that is, one not already in the learner's repertoire and, therefore, likely to conflict with the existing intrinsic dynamics. Given the two types of repertoire that exist before learning, the required relative phase is set to 90° for initially bistable people and to 135° for those with tristable dynamics. Performance feedback is provided during the learning phase, but not in the scans. How do learners handle the new learning tasks? Regardless of whether one is initially bistable or tristable before learning, the mismatch between the produced and required relative phasing pattern diminishes, giving rise to an increase in accuracy of the produced pattern [59]. Stability, not just error, is also a key factor. Whereas learning 90° is accompanied by a significant increase followed by a significant decrease in the variability of performed relative phase, no such changes occur when learning 135° [60].

Scanning probes of the behavioural repertoire after learning reveal what is going on. Initially, bistable learners now exhibit three wells: a new well at the learned pattern (90°) now appearing along with the two that existed prior to learning (figure 3*b*). Learning has reorganized the entire landscape, the initially bistable coordination dynamics becoming tristable. Because the transition from bistable to tristable dynamics is accompanied by changes in stability, we refer to this mechanism of change as the *bifurcation route* to learning [58,61]. In the case of initially tristable learners confronted with the novel task of learning a bimanual phasing pattern of 135°, all that happens is that the original well at 90° shifts towards the just-learned pattern. As a result, learning takes the form of gradual change: the learner's attractor landscape is altered not in terms of number of attractive states (which does not change), only in terms of its layout (figure 3*d*). The overall attractor landscape is not altered qualitatively, a mechanism we refer to as the *shift route* to learning.

On the one hand, the bifurcation route generates qualitative change in an initially bistable behavioural repertoire by adding new stable patterns. Bifurcation is thus a dynamical mechanism for *novelty*. The sudden creation of a new attractive state in the landscape of the coordination dynamics is a reflection of the principle of *selection via instability* [62]. On the other hand, the shift mechanism generates smooth adaptive change. The initial behavioural repertoire is already multi- (here tri-) stable and learning takes the form of gradual change: the learner's dynamic landscape is altered not in terms of the number of attractive states but only in terms of its layout. As a result, behaviour shifts gradually in the direction of the to-be-learned pattern without any instability or gain in stability. Selection of a new behavioural pattern occurs in this case through an effort to match new environmental requirements according to a principle of *selection via matching*.

How humans learn is contingent: depending on the individual's initial repertoire, adaptive changes are governed by a shift mechanism or a bifurcation mechanism. Results show that the bifurcation route leads to greater persistence of a learned behaviour in memory, whereas the shift route, though more flexible, is prone to forgetting [63]. Far from being separate, the two routes depend on each other and may be said to constitute two successive phases of the learning process. The bifurcation mechanism is nevertheless primary. A possible reason is that a minimum level of multistability (attained through the bifurcation mechanism) is needed for learning to evolve gradually through the shift route.

Why is it that some people show up tri- or in general multistable? If our interpretation is correct, then learners who exhibit a tristable initial repertoire must have been bistable at an earlier time: tristability (as we have shown) springs from a basic bistability in the coordination dynamics. Bistability and bifurcation (dynamic instability) may be seen as the primordial cornerstones upon which learning is grounded, the basic ingredients for the sudden emergence of new patterns. Only later does the shift mechanism kick in giving rise to gradual behavioural change. Why the primacy of bistability and bifurcation? As a precursor to tristability, bistability sets the limits of what's possible and what is prohibited. Bistability is primitive: it establishes the space within which learning (and perhaps changes on other timescales as well) can occur. Once established, the multistable dynamics plays out in this space—the space of states defined by coordination variables that capture the interaction or coupling among the parts and processes of goal-directed complex systems.

### (g) Step 7: beyond multistability—bring on metastability

As we have seen, brain circuits can become unstable leading to the emergence of novel coordinative states. Multistable coordination dynamics confers a capacity on the brain to lock in to one of several available patterns. Locking in and switching capabilities can be adaptive and useful, or maladaptive and harmful.

**Figure 4.**
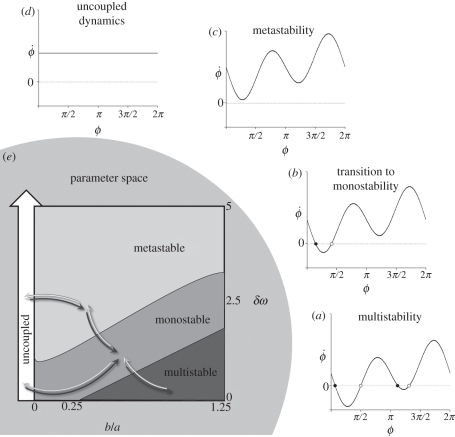
(*a*–*d*) Trajectories of the extended HKB model of coordination dynamics (equation (3.2)) illustrating how metastability (*c*) emerges from multistability (*a*) as a control parameter (*b*/*a*) changes. For (*a*–*c*) the coupling is fixed and *δω* is varied. The case of no coupling (*d*) is shown for comparison. (*e*) The phase diagram or parameter space of the extended HKB model showing the range of behaviours possible as a function of coupling strength (*k* = *b*/*a*) and broken symmetry (δω). Possible paths in parameter space are illustrated by arrows. See text for discussion.

Another kind of mechanism called *metastability* is becoming recognized as an important dynamical mechanism for understanding brain and behavioural coordination. Etymologically, ‘metastability’ comes from the latin *‘meta’* (beyond) and *‘stabilis’* (able to stand). In coordination dynamics, metastability is not just a word. It is the simultaneous realization of two competing tendencies: the tendency of the individual components to couple together and the tendency for the components to express their independent behaviour. In coordination dynamics, metastability corresponds to a regime near a saddle-node or tangent bifurcation in which stable and unstable coordination states no longer exist (e.g. in-phase coordination where the relative phase between interacting components lingers at zero), but attraction remains to where those fixed points used to be. This gives rise to a dynamical flow consisting of phase trapping and phase scattering. Early on metastability was identified in the extended HKB model of coordination dynamics at a behavioural level [64], but was soon seen as an important mechanism for brain coordination [65–70]. Because many brain processes are governed by periodic, often oscillatory dynamics [71–73], the coordination variable coupling the oscillations together is the relative phase, *ϕ*. A fixed point of the coordination variable thus represents a steady-phase and frequency relationship between the oscillatory components or phase-locking (figure 4).

To illustrate formally the connection between multistability and metastability, the flow of the coordination dynamics across a range of values of *δω* is presented in figure 4*a–c* for a fixed value of the coupling parameter, *k* = *b*/*a* = 1, where *a* = 1 and *b* = 1. Stable fixed points (attractors) are represented by filled circles and unstable fixed points (repellors) as unfilled circles. Note these fixed points refer to specific values of the coordination variable, *φ*. The flows shown in figure 4 illustrate four parameter regimes under the influence of the symmetry breaking term *δω* in equation (3.2)*.* In figure 4*a*, the system is multistable, here bistable. For the system to settle in, two stable fixed points (filled circles) constitute the alternatives. Of the two, which one is observed depends on the initial conditions and the size of the basin of attraction. In figure 4*b*, the intrinsically less stable anti-phase state disappears but the stronger attractor near in-phase is still present as well as its repellor partner (open circle). Figure 4*c* shows the metastable regime. The flow no longer intersects the *x*-axis where the fixed points are located: all the fixed points have disappeared. For the sake of completeness, figure 4*d* shows the flow where the components are uncoupled, behaving independently according to their intrinsic dynamics.

What does coordination behaviour look like in the metastable regime? Although all the fixed points have vanished, a key aspect is that there are still some traces of coordination, ‘ghosts’ or ‘remnants’ of where the fixed points once were. Despite the complete absence of phase-locked attractors, the behaviour of the component parts in the metastable regime is not totally independent. Rather, coordination takes the form of dwellings (phase gathering) near the remnants of the fixed points and phase scattering, where the individual components act quasi-independently, expressing their autonomy. In the metastable regime, successive visits to the remnants of the fixed points are intrinsic to the time course of the network, and do not require any additional sources of input. The time the system dwells in each remnant depends on two factors: the degree of asymmetry among the components (longer dwelling for smaller asymmetry) and the strength of the coupling (longer dwelling for larger values of *a* or *b*). Although discovered in a simple system,^4^ such a basic mechanism provides a powerful means to instantiate the flow of thinking and perceiving and moving as ‘stationary transients’ of coordination in neurocognitive networks [9,65–70,75].

The flows shown in figure 4*a–d* are just singular examples of coordination patterns that arise in a much bigger space. The corresponding parameter space or phase diagram is shown in figure 4*e* illustrating: (i) similar behaviour—such as multistability—for a range of parameter values (hearkening back to our earlier discussion of degeneracy and functional equivalence) and (ii) qualitatively different behavioural patterns as parameters cross critical parameter values that lie along boundaries, e.g. separating multistability and monostability. The system affords many paths in many directions. If you are coordinating your body, then you make the path by walking. If you are coordinating your brain, then you make the path by thinking. The rich variety of coordinations possible—whether you are monostable, multistable, metastable or even not coordinated at all—depends on where the system (on all levels) lives in the parameter space of its coordination dynamics and how it moves in it. This message is beginning to be appreciated in neuroscience [14,19,21].

Does the brain make use of such a principle? The classical view of phase-locked synchronization prescribes that each recruited element loses its intrinsic behaviour and obeys the dictates of the assembly. In theories that propose large-scale integration through phase synchronization, for example, the expression of local activity can exist only when a given area is not enslaved into an assembly [76,77]. In the metastable brain, the activity of individual elements obeys neither the intrinsic dynamics of the elements nor the dynamics dictated by the assembly. A delicate balance between the two poles of integration (coordination between individual elements in transiently synchronized ensembles) and segregation (expression of individual behaviour in diverging neural ensembles) is thus achieved [65–69,78,79]. This design plays out in space and time, with ensembles of various sizes coming together and disbanding incessantly [29,30].

## 4. Why metastability?

At the beginning we asked ‘why multistability’? From figure 4*e*, multistability corresponds to a regime where the components are not too different and the coupling is strong relative to metastability which can be seen even for very weak coupling. It is tempting to see metastability as a necessary step towards mono- and multistability. There are several reasons why metastability may be proposed as a candidate principle of coordination for brains and cognitive systems:

— metastability accommodates the coordination of heterogeneous elements (e.g. brain areas having disparate intrinsic dynamics; brain areas whose activity is associated with the movement of body parts or events in the environment),
— although reminiscent of a multistable regime with attractors, a key difference with metastability is that transitions are not actively induced. No disengagement mechanism is required. Neither stochastic noise nor energy inputs in the form of parameter changes are needed in order to switch from one state to the other. Metastability allows even the lowliest of nervous systems to flexibly browse through a set of possibilities (tendencies of the system) thereby avoiding getting stuck in stationary states,
— metastable brain theory favours no extremes, e.g. reflexive versus intrinsic, integrated versus segregated, local versus global. Rather, it reconciles them, and
— metastability is an expression of the full complexity of the brain. Measures of complexity reach a maximum when the balance between segregative and integrative forces is achieved [79]. Note, however, that such measures are based upon stationarity assumptions whereas metastability in coordination dynamics is literally a ‘stationary transient’.

In short, metastability guarantees that the living brain (and complex, goal-directed systems in general) never finds itself frozen for any length of time in a particular coordination state: no energy barriers need to be crossed to visit self-organized metastable tendencies. For this reason, it seems likely that natural selection has latched on to this aspect of self-organization, favouring metastability as necessary for adaptive behaviour [9,10,23].

## 5. The origins of metastability: coupling and complementarity

Multistability is said to offer a powerful and coherent framework for addressing the issue of *binding* or coupling. In metastability, the strong hierarchical coupling between parts and processes is reduced leading to a looser, more flexible form of function. Figure 4 allows us to see how both views may be reconciled within the framework of coordination dynamics. What remains to be addressed is the origin of metastability. Consider figure 4 in reverse, from panel (*d*) to (*a*), and ask a simple question. If the components are uncoupled, how do they get coupled? Where does the coupling come from?

The word ‘coupling’ is used in many scientific contexts: What do we mean by it? Although the sources of coupling may be manifold and ‘coupling’ often appears in explanations (usually of complicated interactions), the word itself is seldom defined. Here, we define coupling after Root-Bernstein & Dillon [80] as ‘the non-random linking between two or more processes’.

Notice that the parts and processes that are coupled according to the governing dynamics of, for example, equation (3.2) are substitutable. The law is universal though its mechanistic manifestations are specific. In our example, the elements correspond to oscillators and the processes concern oscillations: this is convenient because oscillations, which occur on many levels, appear to be the natural language of the brain and cognition [9,71,81]—and according to some, nature too [82].

To cut to the chase, Root-Bernstein proposes that *complementarity* creates the coupling necessary for non-equilibrium systems to form providing a mechanism for the appearance of novel emergent properties, as in self-organization. Remarkably, as in the foregoing discussion of metastability, complementarity—through the eyes of Root-Bernstein & Dillon [80]—sees evolution as a network composed of alternating periods of integration (as molecules and molecular aggregates merge) and divergence (as molecules and aggregates undergo variations). Just as the Weiss criteria (variance of the whole is less than the sum of the variances expressed by the individual parts) have been used to describe the properties of synergies [23], so Root-Bernstein & Dillon use it to describe the formation of sub-assemblies or aggregates. If complementarity provides a generalized mechanism for coupling, then how does this play out here? Concretely, how does the ‘uncoupled dynamics’ of figure 4*d* transform into the metastable tendencies of figure 4*c* and beyond? Logically—after Aristotle's metaphysics [83] and the philosophy of the complementary nature—one has to inquire what the complement of total independence is, as in independent, uncoupled parts. The parts cannot exist in a vacuum. The answer, surely, is integration and the integrated whole: segregation and integration, individuals and collective, parts and wholes are complementary pairs—considered the basis of reality [11,84,85]. Notice that complementarity cannot realize integration and segregation as pure (idealized) states: metastable tendencies require component differences (*δω* ≠ 0; therefore, broken symmetry) and the weakest of coupling (figure 4*c*,*e*). Individualist tendencies for the diverse parts to express themselves coexist with coordinative tendencies to couple and cooperate as a whole.

And now what happens? If the parts are sufficiently different and the coupling is strong enough, then a transition occurs: equilibria emerge spontaneously. Metastability crosses the boundary into monostability (figure 4*c* to *b*). On the basis of evidence and argument, Root-Bernstein & Dillon [80] propose that *homeostasis* emerges from complementarity. Coordination dynamics shows that it cannot be otherwise. Given a system of heterogeneously connected, heterogeneous elements, i.e. where symmetry is broken, coupled by virtue of complementarity, even multiple equilibria can arise if the coupling is strong enough (figure 4*a*). Through coupling and broken symmetry metastability forms leading on to multistability. If you follow the self-organizing route (§3*d*) metastability arises from multistability. If coupling is based on complementarity (which reaches all the way down to the complementary pairs of quantum mechanics), then metastability arises from complementarity and multistability arises from metastability.

## 6. Postscript

It is not unusual for scientists and artists to draw a dichotomy between brain and mind. For example, Henry Miller (1891–1988), the American writer and painter, remarks that ‘nothing happens in the brain except the gradual rust and detrition of cells’. ‘In the mind’, however, ‘worlds unclassified, undenominated, unassimilated, *form, break, unite, dissolve and harmonize ceaselessly*’ (emphasis mine). ‘In the mind-world’, Miller continues, ‘ideas are the indestructible elements which form the jewelled constellations of the interior life. We move within their orbits, freely if we follow their intricate patterns, enslaved or possessed if we try to subjugate them. Everything external is but a reflection projected by the mind-machine’ [86 p. 29]. The parallel between Miller's mind and the multi- and metastable coordination dynamics of the brain described here is obvious. Mind and brain are complementary: they share a common underlying dynamics.

In testimony to the US Congress in October 2010, Huda Akil, former President of the Society for Neuroscience, poses what she calls the ‘grand challenge of elucidating neural choreography’. No single focused level of analysis will suffice to understand the brain and its disorders (see also Akil *et al*. [87]) ‘We need to identify the dancers, identify the nature of the dance and uncover how disease disrupts it’. Here, the dancers are (cognitively, behaviourally and neurally relevant) nonlinear oscillations, and the dance is the functional patterns of interaction between them: multi- and metastable coordination dynamics.

## Endnotes

1 Key questions seldom addressed in the multistability literature concern the identification of relevant ‘state’ variables, control parameters, etc. Stability and multistability are common words. But, stability of what? The list can range from ‘stimulus interpretations’ to ‘percepts’ to ‘perceptual states’ to ‘perceptual decisions’ to ‘meanings’ and so forth. On any level of description, the issue concerns what the attractor states are of. This is important because a first step in modelling means mapping relevant variables onto the states of a dynamical system.

2 A significant aspect of the HKB model is that equations (3.2)–(3.4) can be derived from ‘lower levels’: the individual components and their nonlinear interaction [42]. Thus, though the whole is greater than the sum of the parts, HKB showed that the parts (nonlinear oscillations) can be assembled by virtue of a fundamental, nonlinear coupling of biophysical origin [43] to create the behaviour of the whole.

3 I am indebted to K.J. Jantzen for carrying out this analysis and for kindly providing figure 3.

4 In Einstein's famous aphorism, simple but not too simple. For example, equation (3.2) has rather richer dynamics than typical models of frequency-dependent selection in models of evolutionary dynamics (compare e.g. Nowak [74] p. 48 with figure 4).
